# mHealth: Don’t Forget All the Stakeholders in the Business Case

**DOI:** 10.2196/med20.4349

**Published:** 2015-12-31

**Authors:** Carolyn Petersen, Samantha A Adams, Paul R DeMuro

**Affiliations:** ^1^ Mayo Clinic Rochester, MN United States; ^2^ Tilburg University Tilburg Netherlands; ^3^ Broad and Cassel Fort Lauderdale, FL United States

**Keywords:** Internet, mobile, mobile health, app, social media, health care market

## Abstract

Mobile health (mHealth) facilitates linking patient-generated data with electronic health records with clinical decision support systems. mHealth can transform health care, but to realize this potential it is important to identify the relevant stakeholders and how they might be affected. Such stakeholders include primary stakeholders, such as patients, families and caregivers, clinicians, health care facilities, researchers, payors and purchasers, employers, and miscellaneous secondary stakeholders, such as vendors, suppliers, distributors, and consultants, policy makers and legislators. The breadth and depth of the mHealth market make it possible for mHealth to have a considerable effect on people’s health. However, many concerns exist, including privacy, data security, funding, and the lack of case studies demonstrating efficacy and cost-effectiveness. Many American and European initiatives to address these concerns are afoot.

## Introduction

The evolution of the mobile health (mHealth) market reflects citizens’ interest in using mobile tools to manage their health, and a growing emphasis on patient engagement makes mHealth attractive to health care systems. In addition to encouraging patients to engage in low-threshold personal self-management activities, mHealth affords the ability to link patient-generated data with electronic health records that incorporate various forms of clinical decision support systems. In addition to patients, care providers, and researchers, there are other stakeholders (including health plans, government payors, pharmaceutical and device manufacturers, platform/app providers and regulators) that have an interest in – and potentially significant influence over – the development of mHealth.

Most studies on mHealth have focused on the development and uptake of mobile applications [1]. These often relate to the effects of patients’ mHealth use for condition management or examine the potential influence on care delivery and related costs. Other aspects of these applications have received less attention. We therefore give a quick overview of the primary mHealth stakeholders and then identify key issues that currently inhibit more widespread use of applications and platforms in health care or for health-related purposes. We then look at how governments are trying to change this through regulatory processes and point to a number of points that need to be addressed in future mHealth research.

## Stakeholders in mHealth

Much has been written about mHealth’s potential to transform health care, regulations governing mHealth, particularly the regulation of mobile medical applications, and regulatory effects on technology development. We conducted a quick scan stakeholder analysis based on the framework of the health policy context of developed nations used in comparative health policy analysis [2]. Affected stakeholders include:

 1. Patients: Patients are key stakeholders, using mobile devices to access health records and lab tests, and make appointments. They can participate in their care in the emerging patient-centered health care models, potentially experiencing improved care and fewer medical errors.

2. Families and caregivers: Families and others responsible for patients’ care seek improvements in care delivery and care coordination, reduced medical errors, and more efficient management of their loved one’s care.

3. Clinicians: Many clinicians appreciate the flexibility of mHealth devices and seek to improve care by accessing patients’ records, utilizing computerized physician order entry, and prescribing medications electronically. They must balance costs, security and ease of use.

4. Health care facilities: Hospital and health systems, ambulatory surgery centers, long-term care facilities, home health agencies, other ancillary providers, and community group homes seek improvements in operational efficiency, reductions in the cost of patient care delivery, the ability to facilitate quality measurement, and expanded reporting capabilities.

5. Researchers: Researchers may use mHealth to generate more and potentially better data for use in clinical trials, comparative effectiveness research, and other areas.

6. Policy actors: Policy makers and legislators may gain better data from which to make decisions and facilitate the development of aligned incentives for the stakeholders through use of mHealth.

7. Payors and purchasers (including health insurers): Payors and purchasers, including self-insured employer groups, look to mHealth to improve health outcomes, provide more readily available data, achieve greater efficiencies, and reduce medical errors.

8. Employers: Employers would like mHealth technologies to contribute to greater quality of care in a more cost-effective manner for their employees, for example through wellness programs, as well as improve patient care delivery and reduce absenteeism.

9. Additional stakeholders: Vendors, suppliers, distributors, small-to-medium enterprise app developers and consultants could potentially develop business via mHealth technologies, and major platform providers also benefit from these developments. The diversity of business models coming from the various players also influences the mHealth market and thus user expectations, regulatory processes, etc.

## Mobile Health Market

The scope of the mHealth market, projected to grow through the rest of the decade, foreshadows the possibilities. The connected devices market has been estimated at US$16.4 billion by 2018 [3], nearly 100 million wearable remote monitoring devices are expected to ship through 2019 [4], and the mHealth market is predicted to reach US$49 billion by 2020 [5]. mHealth will grow, too, in terms of users, with 3 million patients to be monitored remotely by 2016 [6] and 50% of an estimated 3.4 billion smartphone users to have downloaded an app by 2018 [7].

Mobile health is already a reality. Twenty-seven percent of US broadband users use at least one connected health device [8], and 25% of US citizens track personal health measures using a wearable fitness device (e.g., a smart watch) or an mHealth app [9]. Wireless baby monitoring devices that measure an infant’s respiration, position, and other characteristics are available [10]. Patients have even begun developing apps for medical needs not addressed by the commercial market (e.g., remote blood glucose monitoring of children) [11].

## mHealth User Expectations

Both patient and care providers believe mHealth has the potential to improve health. In an August 2014 survey [12] of 1,102 patients and 1,406 health care professionals, including 827 doctors, respondents shared several expectations:

1. Patients (84%) and physicians (64%) think technologies such as smartphones are appropriate for diagnosis

2. Patients (64%) and physicians (63%) would use smartphones in blood tests if possible

3. Patients (42%) and physicians (40%) hesitate to use digital technology due to privacy concerns

Providers see value in the use of patient-generated data for agenda-setting, self-case assessment, and identification of barriers that patients face in managing their health [13]. Providers also demonstrate confidence in mobile devices through their own use of devices; 65% of nurses report using a mobile device for professional purposes at work for 30 minutes daily, and 20% report using a device for 2+ hours daily [14].

Despite the interest in mHealth, health care professionals report several concerns, including privacy, data security, funding, a lack of cases studies demonstrating efficacy and cost-effectiveness, and the need for more research [15]. Providers also worry about the workload resulting from widespread uploading of patient-generated data into electronic medical records and safety issues related to data use [16].

Privacy concerns, in particular, remain a barrier to large-scale adoption of mHealth. Only 30% of apps have privacy policies, and two-thirds of these policies are unrelated to the app itself, addressing rather the vendor or third parties [17]. A 2013 Privacy Rights Clearinghouse study of health and fitness apps noted that user information frequently is shared with third parties without users’ knowledge, often without encryption [18]. Among 43 fitness apps reviewed, 72% had a medium or high risk of privacy loss, with free apps the riskiest. Just 43% of the fitness apps had a privacy policy, of which half were accurate.

## Initiatives to Regulate mHealth

The potential benefits of widespread mHealth use have motivated governments to seek protection for both patients and health care professionals.

### United States Initiatives

Members of Congress have expressed interest in modifying the Health Information Portability and Accountability Act (HIPAA) to support market development while protecting US consumers. Key objectives include:

1. Clarify what vendors must do to comply with HIPAA

2. Publish routine regulatory guidance updates to address technology advances

3. Identify implementation standards

4. Clarify how HIPAA affects encrypted data cloud storage when providers cannot access it

5. Provide assistance for HIPAA compliance

Several US regulatory agencies also seek to facilitate development of a mHealth environment. In September 2013 the Food and Drug Administration (FDA) released guidance on medical mobile apps and their application to wearable devices, and in October 2014 issued guidance on the content of premarket submissions for managing medical device cybersecurity. Previous FDA guidances and draft guidances cover social media and Internet information sharing. The Federal Trade Commission addresses development and use of mHealth and mobile devices through data security regulations. Individual states protect consumers through narrower statutes, such as a California mHealth app initiative [19].

### European Union Initiatives

In early 2014, the European Commission released the mHealth “Green Paper,” a pre-policy document for consulting with Member State stakeholders on 11 issues related to the development and use of mobile applications for health care [20]. It was accompanied by a staff working document on the legal framework regulating the development and use of apps in Europe and its adequacy to address the issues raised by apps considered “lifestyle and wellness” devices [21].

Reports composed by the Advisory Groups for the Horizon 2020 Work programs 2016-17 were released later in the year [22]. Several reports referred to information and communication technologies (ICT) as an important area for investment. While these reports span topics broader than health and health care, ICT’s potential to make a difference in the health and well-being of individuals was a cross-cutting theme in most reports.

These documents provide insight into the European Union’s (EU) strategic (research) priorities for the coming funding period. As a group these documents are optimistic, sharing a “promising ethos” of ICT more generally and mHealth in particular. That is, policy makers at the EU level anticipate the potential of these apps to increase access to primary care and prevention programs, improve quality of life, enable more efficient and sustainable health care, cut costs, and empower patients. The reports recognize that sustainable solutions require that intended users take an early, active role in development processes. The reports also point to the need for a greater role for small and medium enterprises in research and innovation and more insights from the social sciences and humanities in uptake and use evaluation.

## Issues on the Horizon

As the mHealth environment evolves, several additional considerations will need to be addressed to support further development of mHealth, including:

1. Regulation of new products and services such as software as a service

2. Regulation of consumer- and patient-developed devices and apps

3. More research on how other processes that formal regulation (e.g. market mechanisms or industry self-regulation) govern developments in mHealth – especially quality assurance

4. Evolution of privacy and data management regulations for the regulation of commerce

5. Privacy-promoting technologies that allow users to interact with providers and exchange data with confidence.

## Abbreviations

EU: European Union
FDA: Food and Drug Administration
HIPAA: Health Information Portability and Accountability Act
ICT: Information and communication technologies
US: United States

## References

[1] Lupton D (2014). Apps as artefacts: Towards a critical perspective on mobile health and medical apps. Societies.

[2] Blank R, Burau V (2010). Comparative Health Policy. 3rd edition.

[3] MarketsandMarkets, Mobile health apps & solutions market by connected devices (cardiac monitoring, diabetes management devices), health apps (exercise, weight loss, women’s health, sleep and meditation), medical apps (medical reference) - global trends & forecast to 2018.

[4] ABI Research Inc (2014). Foundations Emerge for a Revolution in Remote Patient Monitoring.

[5] Grand View Research (2014). mHealth Market Analysis and Segment Forecasts to 2020.

[6] (2012). Juniper Research Ltd.

[7] research2guidance (2013). Mobile Health Market Report 2013-2017: The Commercialization of mHealth Applications (Vol 3).

[8] Parks Associates A (2014). Nearly 30% of U.S. Broadband Households Own and Use a Connected Health Device.

[9] Pai A 9 percent of US adults do not track health or fitness with devices or apps.

[10] (2014). Rest Devices, Inc.

[11] Linebaugh K (2014). Wall Street J Sep 26.

[12] (2014). WebMD.

[13] Nundy Shantanu, Lu Chen-Yuan E, Hogan Patrick, Mishra Anjuli, Peek Monica E (2014). Using Patient-Generated Health Data From Mobile Technologies for Diabetes Self-Management Support: Provider Perspectives From an Academic Medical Center. J Diabetes Sci Technol.

[14] Wolters Kluwer Health (2014). Wolters Kluwer Health Survey Finds Nurses and Healthcare Institutions Accepting Professional Use of Online Reference & Mobile Technology.

[15] Whittaker Robyn (2012). Issues in mHealth: findings from key informant interviews. J Med Internet Res.

[16] Davidson Emma, Simpson Colin R, Demiris George, Sheikh Aziz, McKinstry Brian (2013). Integrating telehealth care-generated data with the family practice electronic medical record: qualitative exploration of the views of primary care staff. Interact J Med Res.

[17] Sunyaev Ali, Dehling Tobias, Taylor Patrick L, Mandl Kenneth D (2015). Availability and quality of mobile health app privacy policies. J Am Med Inform Assoc.

[18] Privacy Rights Clearinghouse.

[19] State of California Office of the Attorney General (2013). September.

[20] (2014). European Commission.

[21] (2014). European Commission.

[22] (2014). European Commission.

